# Validation of the COVID-19 IgG/IgM Rapid Test Cassette (BNCP – 402 and BNCP402) in a South African setting

**DOI:** 10.4102/sajid.v37i1.431

**Published:** 2022-08-31

**Authors:** Gilad Mensky, Tristan Pillay, Alexander von Klemperer, Merika J. Tsitsi, Michelle Venter, Colin N. Menezes, Sarah A. van Blydenstein

**Affiliations:** 1 Department of Internal Medicine, Chris Hani Baragwanath Academic Hospital, Faculty of Health, University of the Witwatersrand, Johannesburg, South Africa; 2 School of Clinical Medicine, Faculty of Health Sciences, University of the Witwatersrand, Johannesburg, South Africa; 3 Division of Orthopaedic Surgery, Chris Hani Baragwanath Academic Hospital, Faculty of Health Sciences, University of the Witwatersrand, Johannesburg, South Africa; 4 Department of Internal Medicine, Helen Joseph Hospital, Faculty of Health Sciences, University of the Witwatersrand, Johannesburg, South Africa; 5 Division of Infectious Diseases, Department, Internal Medicine, Chris Hani Baragwanath Academic Hospital, Faculty of Health Sciences, University of the Witwatersrand, Johannesburg, South Africa; 6 Division of Pulmonology, Department of Internal Medicine, Chris Hani Baragwanath Academic Hospital, Faculty of Health Sciences, University of the Witwatersrand, Johannesburg, South Africa

**Keywords:** COVID-19, antibody test, South Africa, sensitivity, rapid testing, point of care, serological test, immunoassay

## Abstract

**Background:**

Different diagnostic tools could improve early detection of coronavirus disease 2019 (COVID-19). A number of antibody-based serological point-of-care tests have been developed to supplement real-time reverse transcriptase polymerase chain reaction (RT-PCR)-based diagnosis. This study describes the validity of an antibody test, namely the immunoglobulin G (IgG)/immunoglobulin M (IgM) Rapid Test Cassette^®^ (BNCP – 402 and BNCP402), manufactured by Spring Healthcare Services.

**Methods:**

A prospective cohort validation study was undertaken at Chris Hani Baragwanath Academic Hospital between 16 July 2020 and 12 August 2020. A total of 101 patients admitted as COVID-19 cases under investigation were included in the study. They were divided into two categories depending on time since symptom onset: testing performed within seven days (early cohort) and after seven days (late cohort). The rapid antibody test was compared to the RT-PCR.

**Results:**

Overall, the test has a sensitivity and specificity of 85.2% and 80.0%, respectively, for a combination of IgG and IgM. Sensitivity and specificity of IgG testing alone were 81.5% and 85%. Sensitivity improved for testing with increasing time from symptom onset; however, specifity was not significantly different.

**Conclusion:**

The study data adds to the body of evidence that because of relatively low sensitivity and specificity, there is a limited role for antibody-based point-of-care testing in the acute phase of COVID-19 infection, as was the case with this IgG/IgM Rapid Test Cassette (BNCP – 402 and BNCP402). There may exist a role for such testing in patients recovered from prior COVID-19 infection or in seroprevalence studies; however, additional evaluations at later timepoints from symptom onset are required.

## Introduction

Coronavirus disease 2019 (COVID-19) is caused by the virus severe acute respiratory syndrome coronavirus 2 (SARS-CoV-2).^1,2^ This disease initially presented at the end of December 2019 in Wuhan, Hubei, China.^1,2,3^ In South Africa, the first patient was confirmed to have COVID-19 on 05 March 2020.^4^ At present, South Africa has experienced a number of distinct waves of COVID-19 spread through the population, caused by distinct variants of concern.^5,6^ As such, additional information regarding the role of bedside tests in the diagnosis or population screening for COVID-19 remains valuable to clinicians.

Real-time reverse transcriptase polymerase chain reaction (RT-PCR) is recommended as the gold standard for the detection of SARS-CoV-2 infection based on the identification of viral ribonucleic acid (RNA).^7,8,9,10,11^ In clinical practice, RT-PCR is performed using samples from either oropharyngeal (OP) or nasopharyngeal (NP) swabs.^12^ The sensitivity of these tests is dependent upon viral particles present in the nasopharynx or oropharynx. The SARS-CoV-2 infection can persist, as evidenced by faecal shedding of the virus after OP swabs have become negative.^13^ These factors have led to variable sensitivity of RNA RT-PCR detection of SARS-CoV-2 with higher false negatives, particularly in presymptomatic or late disease.^14,15,16,17,18^ The RT-PCR test is relatively expensive, requires specialised equipment and skilled technicians and may be vulnerable to cross-contamination.^15,19^

Serological testing for human anti-SARS-CoV-2 immunoglobulin M (IgM) and immunoglobulin G (IgG) antibodies has been explored to supplement RT-PCR in the diagnosis of COVID-19.^11,15,19,20^ The human immune response to SARS-CoV-2 virus follows a similar course to that previously described for other *Coronaviridae*, with IgM detection as early as Day 3–5 of infection.^11,19,21,22^ Existing evidence suggests, however, that serology-based testing improves in sensitivity with increasing time from symptom onset, with relatively low sensitivity before seven days.^14,22,23,24^ The sensitivity for IgG remained high up to six weeks after symptom onset.^25^ Early in the pandemic, it was suggested that combining serological tests with RT-PCR may improve case detection rate in early disease over RT-PCR alone.^22,23,26^ However, subsequent data suggests low sensitivity in the clinical setting of early phases of acute COVID-19 infection.^27^

In a bid to expand testing and diagnostic capabilities early in the pandemic, many serology-based POC tests were developed.^26,28^ These were predominantly lateral-flow immunoassays (LFA) for the detection of anti-SARS-CoV-2 IgG and IgM.^26^ The utility of rapid tests may extend to identifying individuals who have already seroconverted; thus, this may have a role in public health planning and response^29^ or through ongoing seroprevalence studies.^11,20,27^

Given their relative low cost, ease of use and rapid test result turnaround, such tests could play important roles in the ongoing South African response to the COVID-19 pandemic.

This study was initially conceived and implemented when there existed limited data regarding the role of such serology in the early acute phase of infection. As such, this study sought to explore the performance of such a point-of-care test (the IgG/IgM Rapid Test Cassette (BNCP–402 and BNCP402), manufactured by Spring Healthcare Services), in a South African setting as compared with the gold standard RT-PCR. This test is a qualitative lateral-flow chromatographic immunoassay in human whole blood, serum or plasma specimens.^25^

## Methods

A prospective cohort validation study was performed. Data were collected between 16 July 2020 and 12 August 2020, until 101 tests were performed. The study took place at Chris Hani Baragwanath Academic Hospital in Soweto, Gauteng, South Africa. The study included all inpatients, either suspected of COVID-19 (under investigation) as well as COVID-19-infected patients and presumed noninfected patients. A valid RT-PCR result was required, the result of which was taken to be the gold standard. Viral RNA RT-PCR-based tests were performed on swabs taken from the nasopharynx. The RT-PCR was performed by trained health care staff in accordance with standard facility operating procedures. Patients 18 years or older were recruited. Participants were included in the following categories:

- Positive control group
  - Laboratory confirmed cases of COVID-19 by RT-PCR.
  - Time from symptom onset is > 7 days.
- Negative control group
  - Whilst in an ideal situation, these would be young healthy volunteers, given the resource scarcity of RT-PCR testing at the time of study, this cohort consisted of persons under investigation who were excluded from having COVID-19 clinically and by laboratory measures but may be ill with other disease.
- Indeterminate group under investigation (early cohort)
  - Persons under investigation for whom a NP sample has been taken and sent for laboratory diagnosis, but no result is present at the time of recruitment.
  - Time from symptom onset is < 7 days.
- Indeterminate group under investigation (late cohort)
  - Persons under investigation for whom a NP sample has been taken and sent for laboratory diagnosis but no result is present at the time of recruitment.
  - Time from symptom onset is > 7 days.

The sample size in order to accurately calculate the negative predictive value given the manufacturer specified sensitivity and specificity for IgG:

- Positive control cohort: *n* = 45
- Negative control cohort: *n* = 5
- Undetermined cohort (early): *n* = 25
- Undetermined cohort (late): *n* = 25

Following informed consent, clinical data was captured, namely age, symptoms and duration of symptoms. The rapid test was then performed following manufacturer instructions. This required either the use of a blood sample already collected in an acid citrate dextrose tube or a finger prick test. The patient was informed of the results of the test and appropriately counselled.

The data obtained was centralised in an anonymised Excel spreadsheet and updated daily. Study data were collected and managed using REDCap^®^ (Research Electronic Data Capture) electronic data capture tools hosted at the University of the Witwatersrand.^30^

Data were reported as a percentage, with 95% confidence intervals (CI) unless otherwise specified. For calculation and comparisons of sensitivity and specificity, receiver operating characteristic (ROC) curves were calculated. For comparison between tests, area under the curve (AUC) of the ROC was calculated and compared using the formula:


$$Z=\frac{\left|Area_{1}-Area_{2}\right|}{\sqrt{SE_{Area1}^{2}+SE_{Area2}^{2}}},$$ [Eqn 1]

where SE = standard error of the area. A two-tailed *p*-value was then calculated from the normal cumulative distribution. *P*-values of < 0.05 were considered significant. Further software used in the statistical calculations included GraphPad Prism^®^ (v9.0.2, GraphPad software). The primary endpoints were categorical and hence non-parametric statistical tests were used. The chi-squared and Z test was used to compare the results according to IgG, IgM and time groups.

## Results

**FIGURE 1:**
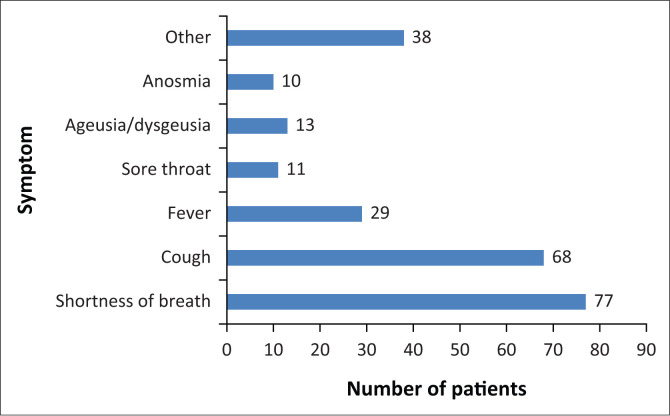
Common presenting complaints of patients under investigation of COVID-19.

The mean time from onset of symptoms to time of testing was 11.25 days (9 h to 38.4 days). A total of 46 men and 55 women were included in the study who ranged in age from 18 to 86 years of age. Interestingly, only 10.0% of patients could positively identify contact with a person under investigation (PUI) or confirmed COVID-19 positive individual. The 90.1% of patients were symptomatic on presentation, most commonly with dyspnoea (Figure 1). All asymptomatic patients were found to be RT-PCR positive for COVID-19.

Patients who presented with ‘other symptoms’ included chest pain (*n* = 16), myalgia (*n* = 8) and gastrointestinal symptoms (*n* = 5).

Of the 101 patients included for testing, 81 had laboratory-proven SARS-CoV-2 infection as evidenced by positive RT-PCR results. Twenty patients were RT-PCR negative for SARS-CoV-2. Using these gold standard test results as true positive and true negative cases, respectively, the performance of the lateral flow antibody (LFAB) could be assessed in point-of-care diagnosis of COVID-19 (Table 1).

**TABLE 1:** Relative performance of antibody lateral-flow assay in detecting SARS-CoV-2.

Statistical measure/index	RT-PCR	IgG	IgM	Combined (IgM or IgG)
True positive	81	66	33	69
True negative	20	17	19	16
False positive	0	3	1	4
False negative	0	15	48	12
		101	101	101
Sensitivity (%) 95% CI	GS	81.5% 71.7–88.4	40.7% 30.7–51.6	85.2% 75.9–91.3
Specificity (%) 95% CI	GS	85.0% 63.7–94.8	95.0% 76.4–99.7	80.0% 58.4–91.9
PPV (%)		95.7	97.1	94.5
NPV (%)		53.1	28.4	57.1

GS, gold standard; NPV, negative predictive value; PPV, positive predictive value; RT-PCR, reverse transcriptase polymerase chain reaction 95% CI, 95% confidence interval; IgG, immunoglobulin G; IgM, immunoglobulin M.

Sensitivity, specificity, negative and positive predictive values are calculated with RT-PCR as gold standard.

Compared with the gold standard RT-PCR, sensitivity of IgG alone (81.5%, IQR: 71.7% – 88.4%) and a combination of either IgG or IgM ( 85.1%, IQR: 75.9% – 91.3%) were significantly higher than the sensitivity of IgM testing alone (40.1%, IQR: 30.7% – 51.6%, χ^2^ of sensitivity IgG vs IgM vs combined = 46.17, degree of freedom (*df*) = 2, *p* < 0.0001). The specificity of all three measures were similar between three categories namely: (1) IgG alone (85.0%, IQR: 64.0% – 94.8%); (2) IgM alone (95.0%, IQR: 76.4% – 99.7%); and (3) a combination of IgG and IgM (80.0%, IQR: 58.4% – 91.9%, χ^2^ of specificity IgG vs IgM vs combined = 2.019, *df* = 2, *p* = 0.3644).

Furthermore, using the ROC analysis at the AUC was significantly greater in the group of data investigating IgG alone compared with that of IgM (AUC = 0.83 vs 0.68, Z = 1.96, *p* = 0.05), meaning that IgG alone performed better as a diagnostic test than IgM alone. There was no difference between either IgG alone (Z = 0.08, *p* = 0.93) or IgM alone (Z = 1.81, *p* = 0.07) compared with a combination of the two.

It has been shown that the time from symptom onset can affect the sensitivity and specificity of serology-based tests such as the lateral-flow assay used^4^, likely because of time taken to peak production of IgG and IgM as part of the immune response. For this reason, comparisons of subpopulations who presented either before (early) or after seven days (late) from symptom onset were undertaken.

**FIGURE 2:**
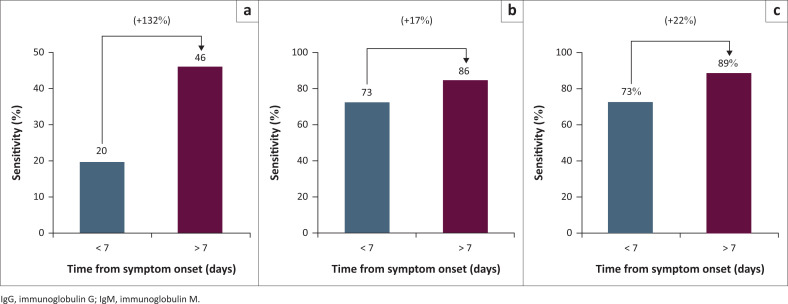
IgG/IgM Rapid Test Cassette (BNCP–402 and BNCP402) – Comparing the sensitivities of (a) IgM, (b) IgG and (c) combined and how they changed with time.

When comparing the early and late cohorts, no statistically significant difference was noted between time periods in the sensitivity of either IgG alone (Figure 2, 73.3% [48.1% – 89.1%] vs 85.0% [74.3% – 92.6%], or a combination of IgM and IgG (Figure 2, 73.3% [48.1% – 89.1%] vs 89.3% [78.5% – 95.0%]. While IgM sensitivity testing showed a trend in improvement from the first seven days, this was not significant (20.0% [7.0% – 45.2%] vs 46.4% [34.0% – 59.3%], Z = 1.85, *p* < 0.06).

**FIGURE 3:**
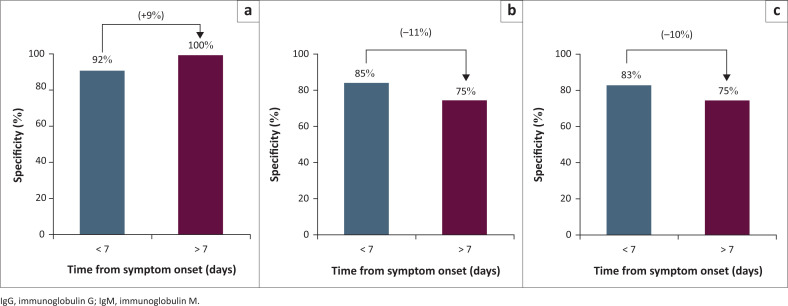
IgG/IgM Rapid Test Cassette (BNCP–402 and BNCP402) – Comparing the specificities of (a) IgM, (b) IgG and (c) combined and how they changed with time.

There were no statistically significant differences in the specificity (Figure 3) of IgG alone (*p* = 0.31), IgM alone (*p* = 0.40) or combined IgM and IgG (*p* = 0.65) between early and late cohorts.

## Discussion

The COVID-19 pandemic has created a significant burden on the health care system in South Africa.^31^ While point-of-care testing with rapid assays remains an important avenue for the diagnosis of COVID-19 infected patients,^32,33^ the role of serological tests as opposed to other rapid testing modalities is now understood to be more limited than was evident early in the pandemic.^27^

This study adds to the body of evidence that such serological testing has a limited role in the acute diagnosis of COVID-19,^14,27^ which is reflected in current guidelines.^34,35^ It should be noted that all patients included in this study were hospital inpatients, and as such it is possible to comment only on the efficacy of the rapid test in this setting.

As of March 2020, the United States Food and Drug Administration requires a sensitivity and specificity of 90% and 95%, respectively, for SARS-CoV-2 diagnostic testing. Other national regulatory bodies have similar high standards of accuracy.^28,36,37^ Relevant to the South African setting, the South African Health Products Regulatory Authority (SAHPRA) requires that serological tests have a sensitivity of > 85% and specificity of > 95%, as compared to the gold standard RT-PCR.^38,39^ The manufacturer-specified standards of the tests used met this criteria under controlled conditions (see Online Appendix 1). Importantly, in an onsite assessment of performance in the hands of clinicians, lower sensitivity and specificity can be found than this, particularly in the first seven days after symptom onset. In particular, the wide variance of test specificity that is observed would make interpretation of a negative result difficult.^40^ As such, this assay is not an adequate test in isolation for diagnostic purposes in the acute setting (i.e. before 14 days post symptom onset). Importantly, the SAHPRA specifications are for the recommended use in patients who are in the maximal IgG production window between 14 and 44 days post symptom onset, which is beyond the scope of this current study.^38,39^ It should, however, be noted that real-world performance of diagnostic testing often can differ from what is seen under more controlled conditions. Indeed, the sensitivity and specificity recorded in the current study are superior to the on-site performance estimates of some similar tests, particularly during the early period of interest.^37,41^

At this stage in the pandemic, the rapid antibody test is likely only useful as a seroprevalence tool within a community to determine level of immunity, either post natural infection or resulting from vaccination. This may be best implemented in an epidemiological seroprevalence or surveillance setting.^11^ Any serology-based rapid tests being considered for use in such a setting should be validated according to guidelines using patients with a median time from symptom onset of 33 days.^38^

Interpretation of such serological tests is also further complicated by the vaccine status of the patient undergoing testing. With the roll-out of a national vaccination program, a significant proportion of the population now has exposure to COVID-19 vaccines.^42,43^ At present, such point-of-care testing should not be recommended for the evaluation of neutralising antibodies and vaccine efficacy; however, an immune response elicited by vaccination may affect the specificity of the IgG/IgM Rapid Test Cassette.

## Conclusion

In the population group tested, the IgG/IgM Rapid Test Cassette (BNCP–402 and BNCP40) had a lower sensitivity and specificity as compared with the gold standard RT-PCR than would allow for reliable diagnosis of acute COVID-19. The data in this study support the general guidelines listed by the Centers for Disease Control and Prevention and SAHPRA suggesting that antibody testing does not have a place in the acute diagnosis of COVID-19. These results do not speak to the rapid tests’ sensitivity and specificity in patients who have recovered from prior COVID-19. Further study would be recommended in a patient cohort further from symptom onset, as there may still be a role for such a point-of-care test in such patients or in an epidemiological surveillance role.

## Limitations

This study only included in-hospital patients, and unfortunately, details of symptoms and severity were not captured.

This study was undertaken during the first and second waves of COVID-19. South Africa is currently in the fifth wave, and the landscape has changed significantly in that the population has access to vaccination against COVID-19, and a proportion of people have prior infection. At the time that this study was undertaken, antibody testing was not as well established in diagnostic laboratories compared to now.
